# The efficacy of a β-hydroxy-β-methylbutyrate supplementation on physical capacity, body composition and biochemical markers in elite rowers: a randomised, double-blind, placebo-controlled crossover study

**DOI:** 10.1186/s12970-015-0092-9

**Published:** 2015-07-30

**Authors:** Krzysztof Durkalec-Michalski, Jan Jeszka

**Affiliations:** Department of Hygiene and Human Nutrition, Poznań University of Life Sciences, Wojska Polskiego 31, Poznań, 60-624 Poland; Polish Wrestling Federation, Żelazna 67/73, Warsaw, 00-871 Poland

**Keywords:** β-hydroxy-β-methylbutyric acid, Sport supplements, Training support, Adaptation, Rowing

## Abstract

**Background:**

β-hydroxy-β-methylbutyric acid (HMB) is an interesting supplement in sports. However, literature sources present a limited number of studies that verify the efficacy of HMB intake over a longer time period among endurance athletes. For this reason, the aim of this study was to assess the effect of HMB supplementation on physical capacity, body composition and levels of biochemical markers in rowers.

**Methods:**

Sixteen elite male rowers were administered a 12-week HMB supplementation (3×1 g_HMB_ · day^−1^) and placebo administration (PLA) following the model of a randomised, placebo controlled, double-blind crossover study with a 10 days washout period. Over the course of the experiment, aerobic (maximal oxygen uptake, ventilatory threshold) and anaerobic (anaerobic power indices) capacity were determined, while analyses were conducted on body composition as well as levels of creatine kinase, lactate dehydrogenase, testosterone, cortisol and the T/C ratio. A normal distribution of variables was tested using the paired 2-tailed t-tests; the Mann–Whitney *U*-test or the Wilcoxon-signed rank test were applied for non-normally distributed variables.

**Results:**

Following HMB supplementation, $\dot{\mathrm{V}}{\mathrm{O}}_2 \max$ increased (+2.7 mL · min^−1^ · kg^−1^) significantly (*p* < 0.001) in comparison to its reduction after PLA (−1.0 mL · min^−1^ · kg^−1^). In turn, at the ventilatory threshold, a longer time was required to reach this point (+1.2 min_HMB_ vs. −0.2 min_PLA_, *p* = 0.012), while threshold load (+0.42 W · kg^−1^_HMB_ vs. −0.06 W · kg^−1^_PLA_, *p* = 0.002) and threshold heart rate (+9 bpm_HMB_ vs. +1 bpm_PLA_, *p* < 0.001) increased. After HMB supplementation, fat mass decreased (−0.9 kg_HMB_ vs. +0.8 kg_PLA_, *p* = 0.03). In relation to the initial values after HMB supplementation, the refusal time to continue in the progressive test was extended (*p* = 0.04), maximum load (*p* = 0.04) and anaerobic peak power (*p* = 0.02) increased. However, in relation to the placebo, no differences were observed in anaerobic adaptation or blood marker levels.

**Conclusions:**

The results indicate that HMB intake in endurance training has an advantageous effect on the increase in aerobic capacity and the reduction of fat mass. It may also stimulate an increase in peak anaerobic power, while it seems to have no effect on other indices of anaerobic adaptation and levels of investigated markers in the blood.

## Background

β-hydroxy-β-methylbutyric acid (HMB) – a metabolite of leucine and 2-ketoisocaproic acid – has, for almost 20 years, drawn special attention regarding supplementation support in sports [1–5]. A major advantage of its use suggested in the literature is connected with its anticatabolic action, manifested particularly when there is a high load on the body and when muscle damage is experienced, which may result from the potential effect of HMB on the enhancement of the synthesis or the inhibition of the proteolysis of muscle proteins [2, 5–7]. The observed effect of HMB on the reduction of body mass loss, muscle mass loss, and the degree of cancerous cachexia, as well as slowed neoplasm proliferation, should also be mentioned here [8, 9]. These beneficial effects might be connected with the influence of HMB on the de novo synthesis of cholesterol [5, 7, 10], the expression of insulin-like growth factor 1 (IGF-1) [11, 12], the stimulation of the mTOR kinase pathway [6, 8, 12, 13] or the ubiquitin-proteasome system and caspase activity [7, 9, 14–16]. The possible impact of HMB on the activation of AMPK kinase and Sirt 1, may promote stimulation of mitochondrial biogenesis, higher oxygen consumption, increased efficiency of carbohydrate and fat metabolism, increased lipolysis and fat mass reduction [17, 18].

Studies assessing the effects of HMB in physically active individuals have mainly focused on verifying changes in the state of nutrition, assessing protein synthesis and proteolysis rates, and monitoring hormone levels and selected indices illustrating, for example, the degree of muscle damage and determination of changes in physical capacity [3, 19–22]. Since 1996, studies have been published that claim that HMB uptake may promote advantageous changes in body composition and strength, and reduced levels of muscle damage markers during resistance training [3, 18, 22, 23]. Further, in a meta-analysis by Nissen and Sharp [21], it was found that HMB supplementation for resistance exercise resulted in increased strength and fat-free mass by (net value) 1.4 and 0.28 % per week, respectively, in both trained and untrained individuals.

In contrast, the effect of HMB uptake on physical capacity has rarely been verified in endurance sports. HMB-supplemented cyclists and runners showed an increase in aerobic adaptation, which was assessed by maximal oxygen uptake ($\dot{\mathrm{V}}{\mathrm{O}}_2 \max$), ventilatory threshold (VT), peak oxygen uptake ($\dot{\mathrm{V}}{\mathrm{O}}_2\mathrm{peak}$), time needed to reach $\dot{\mathrm{V}}{\mathrm{O}}_2\mathrm{peak}$, a delay in the onset of blood lactate accumulation (OBLA), and a lower activity of creatine kinase (CK) and lactate dehydrogenase (LDH) [20, 24–26].

However, it’s important to observe that certain studies did not show the effectiveness of HMB in enhancing aerobic capacity [3, 20]. A lack of evidence for the effectiveness of HMB supplementation on changes in body composition, activity of muscle damage markers, or in hormone concentrations was also presented in studies on participants involved in resistance or volleyball training [3, 27–29].

In view of the inconclusive character of the study results conducted to date, and the relatively low number of studies investigating the effectiveness of HMB over a longer period on endurance trained athletes, the aim of this study was to verify the effect of HMB supplementation on physical capacity, body composition and the levels of biochemical markers in elite athletes practicing rowing.

## Methods

### Subjects

The characteristics of the examined group of athletes are given in Table 1. The experiment involved 16 elite male rowers, aged 20 ± 2 years, with a body weight of 87.3 ± 9.8 kg and a height of 187 ± 5 cm. Athletes were members of the Polish National Team in Rowing and/or rowers occupying high positions in national competitions. The criteria for qualifying for the study included, among others, a good state of health, a valid and current medical certificate confirming their ability to practice sports, at least 5 years of training experience, and a minimum of five rowing training sessions per week. The investigations were conducted from 2009 to 2014 at different times of the year. All athletes declared that they had not undergone changes in their lifestyle, characteristics of training, nutrition, or supplementation, and that they were not using any preparations with potential ergogenic effects, other than those supplied by the authors of this study. Moreover, dietary journals were performed every second week, which proved that athletes did not change their dietary habits during the supplementation period.

**Table 1:** Characteristics of the participating rowers (*n* = 16)

	Study group	
Parameter	Means ± SD	Range
Age (yr)	19.5 ± 1.4	17.0 – 22.0
Body mass (kg)	87.3 ± 9.8	69.0 – 104.7
Height (cm)	187 ± 5	176 – 194
FFM (kg)	73.8 ± 6.4	63.9 – 83.4
FM (kg)	13.6 ± 5.3	5.1 –21.6
$\dot{\mathrm{V}}{\mathrm{O}}_2 \max$ (mL · min^−1^ · kg^−1^)	68.1 ± 6.4	58.4 – 75.0
Peak power (W · kg^−1^)	11.6 ± 1.1	9.9 – 13.2
Years training (yr)	8.2 ± 2.8	6 – 10
Number of training sessions per week (session · week^−1^)	8.6 ± 2.7	7 – 10
Number of hours of training per week (h · week^−1^)	16.8 ± 5.9	10 – 24
Energy intake (kcal · kg^−1^ · day^−1^)^a^	56.1 ± 14.2	37.0 – 79.2
Protein intake (g · kg^−1^ · day^−1^)^a^	1.6 ± 0.2	1.3 – 2.0
Carbohydrate intake (g · kg^−1^ · day^−1^)^a^	6.3 ± 1.4	4.9 – 9.3
Fat intake (g · kg^−1^ · day^−1^)^a^	1.7 ± 0.4	1.2 – 2.4

*FFM* fat-free mass, *FM* fat mass; $\dot{V}{O}_2 max$ maximal oxygen uptake

^a^Mean energy and macronutrient intake during the supplementation period

In accordance with the Declaration of Helsinki, all the participants expressed their free and conscious consent to participate in the research procedures. The consent of the Bioethics Committee at Poznań University of Medicine Sciences was obtained for this study (decision no. 584/09 of 18 June 2009).

### Experimental design

#### Characteristics of the administered supplementation

**Fig. 1:**
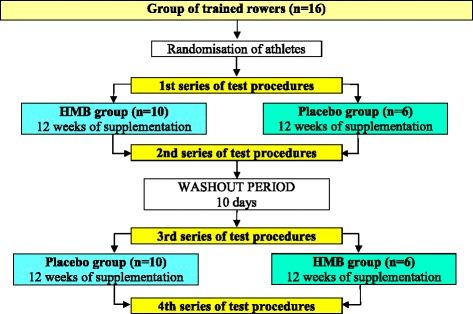
A flow chart of the study design

The effect of HMB supplementation was assessed in randomised, crossover, double-blind tests (Fig. 1). The experimental procedure for each athlete included a 12-week supplementation with an HMB preparation and a 12-week placebo administration. Upon determining experiment qualification, the athletes were subjected to the randomisation procedure (based upon lean body mass) and assigned either to the group receiving the HMB preparation in the first 12 weeks of the trial or to the group receiving the placebo. After 12 weeks, a 10-day washout period was implemented, which was similar in other studies and sufficient given the kinetics of HMB absorption and excretion from the body [24, 30, 31]. After the washout period, a crossover exchange of the preparations administered to the groups was applied.

The experiments were conducted using a preparation of calcium salt of HMB produced by Olimp Laboratories. A single capsule contained 1250 mg of Ca-HMB, which corresponds to 1000 mg HMB. The producer also prepared a placebo preparation containing maltodextrin. The tested group of athletes was administered three capsules of the assigned preparation per day in three doses as follows: upon waking, immediately after training, and before sleep. On non-training days, the participants were instructed to consume one serving with each meal throughout the day. The consumed HMB dose was equivalent to the most commonly recommended intake of 3 g HMB per day [2, 3, 5, 19, 26].

Among all participants, the efficacy of HMB supplementation was assessed using four series of research (each included: evaluation of body composition, aerobic and anaerobic capacity, as well as blood sampling and biochemical analyses), consisting of identical procedures in two cycles separated by a washout period: the tests took place prior to the onset of the intervention (Pre_HMB_ and Pre_PLA_), following the 12 weeks of supplementation with the HMB preparation (Post_HMB_) and the placebo (Post_PLA_). As mentioned in the methods section, all athletes declared that they had not undergone changes in their lifestyle, characteristics of training, or nutrition.

#### Evaluation of body mass and composition

Body weight and height were measured using a WPT 60/150 OW medical anthropometer by RADWAG® (Poland). Body composition was analysed by determining the values of electrical resistance and reactance through bioelectric impedance with the use of a BIA 101S analyser by AKERN-RJL (Italy) and the Bodygram 1.31 computer software by AKERN-RJL (Italy). Body composition was measured strictly following the recommended measurement conditions, i.e. in the morning hours, following overnight fasting, in subjects lying in a supine position, and with the recommended application of measuring electrodes [32]. Athletes were also instructed to abstain from drinking coffee, strong tea, caffeine-containing products, and alcohol for at least 24 h before the test, as well as to refrain from physical exercise for a minimum of 18 h before measurements.

#### Assessment of aerobic capacity

The exercise tests to assess the selected capacity parameters in the athletes were conducted in the morning hours (between 7:00 and 10:30 a.m.), always in the same conditions (temperature of 20–22 °C, relative humidity of 50–60 %) at the Exercise Tests Laboratory at the Department of Human Nutrition and Hygiene, Poznań University of Life Sciences. Prior to each test, the athletes were informed, in detail, of the objective, procedure and methods of the exercise tests. The level of aerobic capacity in athletes was assessed based on the recorded maximal oxygen uptake ($\dot{\mathrm{V}}{\mathrm{O}}_2 \max$) and the VT during a test that involved performing exercise with increasing intensity (+50 W each 3 min) on a Kettler X1 cycloergometer (Kettler, Germany), following recommendations for such tests [33]. During the tests, the respiration indices were recorded using a portable K4b^2^ ergospirometer (Cosmed, Italy) and the COSMED CPET Software Suite (ver. 9.1b, 2010). Moreover, the Cosmed K4b2 system was calibrated prior to each individual test according to the manufacturer’s guidelines.

In this study, maximal exercise was assumed to occur when an increase in load failed to produce an increase in oxygen uptake ($\dot{\mathrm{V}}{\mathrm{O}}_2$) and heart rate (HR). In order to determine the VT, the V-slope method was applied based on an analysis of linear regression for the curve of increasing CO_2_ exhalation in relation to the curve of increasing O_2_ uptake [34].

#### Blood sampling and biochemical analyses

The most widely used markers of adaptation and homeostasis in studies involving athletes were applied in this investigation. The activity of the CK and LDH enzymes, and the concentration of testosterone and cortisol (for calculating the T/C ratio) were assessed based on a quantitative analysis of the blood plasma of the athletes using commercial diagnostic tests. Twenty to twenty-five minutes after the exercise test, blood samples were collected from the athletes from the ulnar vein to two closed-system evacuated test tubes of 2.7 mL, using lithium heparine and sodium fluoride as anticoagulants (Sarstedt Monovette®, Germany). The collected plasma was subjected to further laboratory analyses on the same day. CK and LDH activity were assayed using a standardised colorimetric enzymatic method with a COBAS® 6000 analyser (module c 501, Roche/Hitachi, USA). The concentrations of testosterone and cortisol in blood plasma were assayed by ECLIA electrochemiluminescence using a COBAS® 6000 analyser (module e 601, Roche/Hitachi, USA).

#### Assessment of anaerobic capacity

Anaerobic capacity was assessed using the classical Wingate test on a cycloergometer (Monark 894E, Sweden) following recommendations for such tests proposed by Bar-Or [35]. Seat height was adjusted to each participant’s satisfaction, and toe clips with straps were used to prevent the feet from slipping off the pedals. The primary test was preceded by a 5-min warm-up period of approximately 50 W power, followed by a 5-min break. This was followed by two run-up practices of 3 s, during which the actual test load was imposed to accustom the participants to the resistance. The test lasted for 30 s. External loading was estimated individually at 7.5 % body weight. During the test, the athletes were encouraged to maintain maximum effort. Recorded results included the peak power output (PP), the average power output (AP), and the mean power output (MP), which were analysed using Monark Anaerobic Test Software (ver. 3.0.1, 2009, Sweden). The familiarization session for subjects was not needed, because the rowers were quite familiar the Wingate test, due to previous studies and training procedure.

#### Statistical analysis

All statistical calculations were performed using the Statistica 10.0 package (StatSoft, Poland, 2011). Sample size was determined using previously published data [25, 26]. Basic descriptive statistics were calculated for the tested parameters, and the results are presented as means and standard deviations (± SD) for at least four independent series of measurements. The Shapiro-Wilk test was applied in order to determine whether the random sample came from a population with a normal distribution. The statistical analysis was performed based on the research hypothesis that the HMB supplementation support the physical capacity and body composition regulation in trained athletes. Therefore statistical tests were selected in order to compare the significance of the changes resulting from the HMB supplementation or placebo. Since a crossover design was used in this study and all subjects received both HMB and placebo. The significance of the differences in the mean value of parameters between HMB and placebo were tested using independent samples t-tests in the case of normally distributed variables, or by Mann–Whitney U tests in the case of non-normally distributed variables. Differences in mean values of parameters between baseline (Pre) and post-intervention (Post) were tested by dependent samples t-tests (normally distributed variables) or Wilcoxon-signed rank tests (non-normally distributed variables).

## Results

### Body composition

**Fig. 2:**
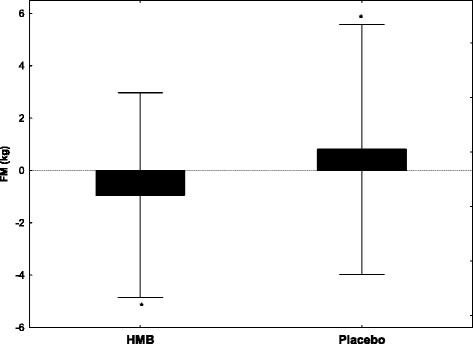
Changes in fat mass after 12-week supplementation of HMB. Values are expressed as mean ± SD. Significant differences compared with placebo (independent samples t-tests) at: * *p* = 0.03

Following the 12-week HMB supplementation, a significant decrease (*p* = 0.03) was recorded in fat mass (−0.9 kg_HMB_), while after the placebo treatment this tissue component increase (+0.8 kg_PLA_) (Fig. 2). Moreover, in both groups, weight loss was observed, although there were no differences between the HMB and placebo. In relation to the pre-investigation value, after HMB supplementation, the changes in body mass were significant (*p* = 0.016), but were associated with a slight (*p* = 0.12) reduction in lean body mass, which significantly decreased by 2.1 kg (*p* = 0.001) after the placebo treatment (Table 2).

**Table 2:** Levels of the monitored indices before and after the 12-week supplementation with HMB preparation and placebo

Research period		PRE_HMB vs. PLA_	HMB	Difference	PLACEBO	Difference
		*p* value^a^		*p* value^b^		*p* value^b^
BM (kg)	Pre	0.925	86.5 ± 9.3	0.016	86.2 ± 9.0	0.063
	Post		84.6 ± 8.1		84.8 ± 8.5	
FFM (kg)	Pre	0.921	72.7 ± 6.3	0.117	72.9 ± 6.1	0.001
	Post		71.7 ± 5.6		70.9 ± 5.7	
FM (kg)	Pre	0.755	13.8 ± 4.4	0.073	13.3 ± 4.7	0.197
	Post		12.8 ± 4.0		14.1 ± 4.3	
$\dot{\mathrm{V}}{\mathrm{O}}_2 \max$ (mL · min^−1^)	Pre	0.681	5794 ± 676	0.240	5897 ± 735	0.067
	Post		5905 ± 709		5723 ± 745	
$\dot{\mathrm{V}}{\mathrm{O}}_2 \max$ (mL · min^−1^ · kg^−1^)	Pre	0.572	67.3 ± 6.9	0.033	68.8 ± 8.0	0.438
	Post		70.0 ± 6.9		67.8 ± 8.5	
Tref (min)	Pre	0.947	17.6 ± 3.1	0.043	17.7 ± 3.1	0.233
	Post		19.0 ± 3.6		18.5 ± 3.7	
Wmax (W)	Pre	0.777	372 ± 52	0.052	378 ± 52	0.625
	Post		397 ± 56		384 ± 60	
Wmax (W · kg^−1^)	Pre	0.621	4.31 ± 0.50	0.039	4.39 ± 0.41	0.234
	Post		4.70 ± 0.55		4.55 ± 0.65	
HRmax (bpm)	Pre	0.962	185 ± 13	0.258	185 ± 9	0.136
	Post		188 ± 9		188 ± 10	
T_VT_ (min)	Pre	0.440	12.5 ± 2.1	0.003	12.8 ± 1.9	0.619
	Post		13.7 ± 2.2		12.6 ± 2.6	
W_VT_ (W)	Pre	0.187	278 ± 41	0.012	294 ± 36	0.310
	Post		309 ± 42		284 ± 47	
W_VT_ (W · kg^−1^)	Pre	0.248	3.24 ± 0.51	0.001	3.42 ± 0.32	0.554
	Post		3.66 ± 0.36		3.36 ± 0.49	
HR_VT_ (bpm)	Pre	0.399	157 ± 9	0.001	160 ± 10	0.598
	Post		166 ± 11		161 ± 9	
CK (U · L^−1^)	Pre	0.865	328 ± 160	0.004	348 ± 169	0.008
	Post		241 ± 103		265 ± 156	
LDH (U · L^−1^)	Pre	0.573	318 ± 36	0.033	326 ± 45	0.002
	Post		302 ± 35		302 ± 39	
Testosterone (mg · dL^−1^)	Pre	0.895	510 ± 202	0.352	510 ± 154	0.569
	Post		552 ± 160		487 ± 160	
Cortisol (mg · dL^−1^)	Pre	0.585	19.9 ± 5.3	0.331	20.0 ± 5.9	0.516
	Post		20.6 ± 5.8		20.8 ± 4.1	
T/C ratio (T · C^−1^*10^−2^)	Pre	0.51	3.39 ± 1.77	0.53	3.36 ± 0.99	0.30
	Post		3.41 ± 1.08		3.03 ± 1.11	
Peak power (W)	Pre	0.615	1003 ± 125	0.027	1028 ± 147	0.853
	Post		1054 ± 116		1024 ± 122	
Peak power (W · kg^−1^)	Pre	0.488	11.65 ± 1.01	0.020	11.92 ± 1.16	0.960
	Post		12.26 ± 0.83		11.91 ± 1.15	
Average power (W)	Pre	0.924	725 ± 87	0.648	722 ± 87	0.660
	Post		720 ± 83		718 ± 82	
Average power (W · kg^−1^)	Pre	0.854	8.36 ± 0.60	0.688	8.32 ± 0.55	0.736
	Post		8.30 ± 0.59		8.28 ± 0.65	
Minimal power (W)	Pre	0.478	503 ± 75	0.095	486 ± 52	0.763
	Post		475 ± 54		491 ± 78	
Minimal power (W · kg^−1^)	Pre	0.465	5.80 ± 0.78	0.119	5.63 ± 0.55	0.821
	Post		5.49 ± 0.53		5.67 ± 0.76	

Values are expressed as mean ± SD

*BM* body mass, *FFM* fat-free mass, *FM* fat mass, $\dot{V}{O}_2 max$ maximal oxygen uptake, *Tref* exercise time before athlete’s refusal to continue exercising, *Wmax* maximum load, *HRmax* maximum heart rate, *VT* ventilatory threshold, *T*_*VT*_ time to VT, *W*_*VT*_ load at VT, *HR*_*VT*_ heart rate at VT, CK creatine kinase, *LDH* lactate dehydrogenase, *T/C ratio* testosterone/cortisol ratio

^a^Depending on the data distribution: independent samples t-tests or Mann–Whitney U tests

^b^Depending on the data distribution: dependent samples t-tests or Wilcoxon-signed rank tests

### Maximal oxygen uptake ($\dot{\mathrm{V}}{\mathrm{O}}_2 \max$)

**Fig. 3:**
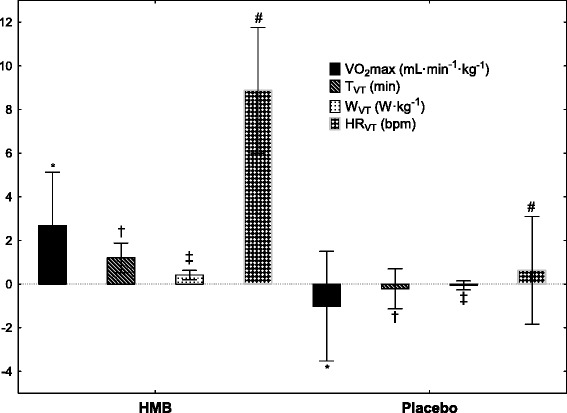
Changes in maximal oxygen uptake and rates at ventilatory threshold after 12-week supplementation of HMB. Values are expressed as mean ± SD. Significant differences compared with placebo (independent samples t-tests) at: *- *p* = 0.03; ^†^ **-** *p* = 0.012, ^‡^ **-** *p* = 0.002; ^#^ **-** *p* < 0.001. $\dot{\mathrm{V}}{\mathrm{O}}_2 \max$: maximal oxygen uptake; T_VT_: time to VT; W_VT_: load at VT; HR_VT_: HR at VT

The analysis of the aerobic capacity indices showed an increase (*p* = 0.03) in $\dot{\mathrm{V}}{\mathrm{O}}_2 \max$ following the 12 weeks of supplementation with the HMB preparation (+2.7 mL min^−1^ · kg^−1^) in comparison to the reduction of aerobic capacity following the placebo treatment (−1.0 mL · min^−1^ · kg^−1^) (Fig. 3). Moreover, in relation to the pre-investigation value, after the HMB supplementation, a significant increase was recorded in $\dot{\mathrm{V}}{\mathrm{O}}_2 \max$ (*p* = 0.03) and exercise time (+1.4 min_HMB_, *p* = 0.04) and maximal load of cycloergometer (+0.38 W · kg^−1^_HMB_, *p* = 0.04), before the athlete refused to continue the test.

### Ventilatory threshold

Analysis of the changes in the recorded markers shows that, following HMB supplementation, time to reach VT (T_VT_: +1.2 min_HMB_ vs. −0.2 min_PLA_, *p* = 0.012), relative threshold load at VT (W_VT_: +0.42 W · kg^−1^_HMB_ vs. −0.06 W · kg^−1^_PLA_, *p* = 0.002), and the threshold HR at VT (HR_VT_: +9 bpm_HMB_ vs. +1 bpm_PLA_, *p* < 0.001) (Fig. 3) increased significantly. In addition, in comparison to the pre-investigation value after the HMB treatment, a significant increase was recorded in T_VT_ (*p* = 0.003), W_VT_ (*p* < 0.001) and HR_VT_ (*p* < 0.001) (Table 2).

### Biochemical blood markers

Thus was no statistically significant differences between the HMB and placebo groups in the tested biochemical markers in the blood. In relation to the initial concentration of the markers assessed, in both groups, significant decreases were recorded in CK activity (CK_POST-HMB_-CK_PRE-HMB_: −87 U · L^−1^, *p* = 0.004; CK_POST-PLA_-CK_PRE-PLA_: −83 U · L^−1^, *p* = 0.008) and LDH activity (LDH_POST-HMB_-LDH_PRE-HMB_: −15 U · L^−1^, *p* = 0.033; LDH_POST-PLA_-LDH_PRE-PLA_: −24 U · L^−1^, *p* = 0.002) after the exercise test with increasing intensity (Table 2).

### Anaerobic capacity

Comparing HMB supplementation to the placebo, no significant differences were observed in the changes in the anaerobic power indices during the Wingate test. However, the analysis of the peak power output showed an increase (*p* = 0.02) following the 12 weeks of supplementation with the HMB preparation (+0.61 W · kg^−1^) in comparison to the pre-HMB period (Table 2).

## Discussion

The findings in this study indicate that a 12-week HMB supplementation in athletes practicing endurance sports influences a reduction of fat mass (*p* = 0.03). Despite the observed lack of differences in the diets of rowers in the course of the experimental procedure, changes in body weight found in both periods were probably caused by a low energy intake, witch concern only some athletes. It may have resulted from the insufficient coverage of high-energy expenditure connected with rowing training. As mentioned in the methodological part, dietary recordings were performed every second week, during the whole study, which proved that athletes did not change their dietary habits during the HMB supplementation and placebo period. Furthermore the potential impact energy intake is significantly reduced by randomised crossover design of the study. Thus, the observed effect of HMB seems considerable, since a reduction of body weight in supplemented athletes was connected with not significant reduction of FM and FFM, while in placebo group FFM reduction was statistically significant (*p* = 0.001). Additionally, despite the supreme importance of aerobic capacity in endurance sports, a significant role may also be played by a high level of anaerobic adaptation, particularly at the start or finish of the race [36, 37]. In this study, in relation to the initial values, only after HMB supplementation was an increase observed in peak power (*p* = 0.02). In turn, in relation to placebo administration after HMB intake, advantageous trends were observed indicating an increase in PP (*p* = 0.06). No differences were found in the case of the other indices of anaerobic capacity of rowers.

However, we would like to highlight here that the aim of the training regime in the investigated group of athletes was not to change body composition or anaerobic adaptation, but to increase their training potential. Probably for this reason, changes in fat-free body mass and power were less important, connected not only with the assumed negative energy balance (suggested by the reduced body weight of athletes), but particularly with the lack of an adequate exercise stimulus for the synthesis of muscle proteins. This may suggest a limited role of HMB, as postulated in the literature, in the activation of, for example, mTOR kinase pathways [6, 8, 12, 13], expression of insulin-like growth factor-1 (IGF-1) [11, 12], or the reduction of activity of the ubiquitin-proteasome system and caspase activity [9, 14–16]. This thesis may be confirmed by the studies, in which individuals supplemented with HMB performed only resistance exercise, stimulating (to a greater extent) an increase in fat-free body mass, which was reported, for example, by Nissen et al. [2] (FFM: +1.21 kg_3.0gHMB_ vs. +0.8 kg_1.5gHMB_ vs. +0.4 kg_PLA_) and Gallagher et al. [19] (FFM: +1.9 kg). In turn, upon HMB supplementation in experiments on volleyball players, in which a key role was played by muscle power, an increase in FFM (+2.3 kg) was recorded, as well as reduction of fat mass by ~0.6 kg_FM_ - comparable to that found our this study [3]. Moreover, in athletes administered the placebo in Portal et al. [3], a slight decrease in FFM (0.1 kg) and an increase of fat mass (3.6 % _FM_) was found. Furthermore, with an increase in FFM in volleyball players, an increase was recorded in peak (1.7 W · kg^−1^_HMB_ vs. 0.4 W · kg^−1^_PLA_) and average power (0.9 W · kg^−1^_HMB_ vs. 0.1 W · kg^−1^_PLA_). These observations may be confirmed by a study by Molinari et al. [38], who recorded increased muscle power (9.2 % _HMB_ vs. 1.7 % _PLA_) in volleyball players using HMB. In turn, in a study on judoists subjected to a 3-day limitation of energy intake (20 kcal · kg_bm_^−1^ day^−1^) a reduction (*p* < 0.05) was recorded for fat mass (−0.85 % _HMB_ vs. 0.2 % _PLA_) only in the group of athletes supplemented with HMB, although no differences were found in indices of anaerobic power between athletes using HMB and placebos [23]. Apart from the negative energy balance, this may have resulted from the fact that HMB supplementation lasted only 3 days, which seems too short to cause significant changes in systemic anaerobic potential. HMB impact on body composition observed also Caperuto et al. [39]. In rats supplemented with 320 mg · kg^−1^ body weight of HMB, carcass fat significantly decreased. Such a large effect was not observed in our study, which we may assume could be related to the dose/response concept. Furthermore, no significant results from the supplemented group when compared to placebo might explain the fact, that HMB supplementation can increase adaptation and promotes metabolic changes in a time-dependent manner [39]. In turn, in the case of endurance sports, changes in FM described in this study seem to be explained by the increase in fatty acid oxidation, as well as lipolysis and insulin sensitivity (e.g., due to the stimulation of activation of AMPK kinase, Sirt1 and the dependent metabolic pathways) [18].

We need to stress here that there is a limited body of literature assessing the efficacy of HMB intake in endurance sports. In terms of the effect of HMB on body composition in runners, Knitter et al. [20], Lamboley et al. [26] and Robinson et al. [25] observed no differences in body composition in athletes who were administered HMB or a placebo. Does that mean that HMB has a definite effect on body composition and anaerobic adaptation only in individuals involved in resistance training? This study was conducted on trained rowers, practicing (first of all) to increase endurance, although their training procedure also included periodical resistance exercise. The recorded results seem to indicate efficacy of HMB supplementation in relation to the advantageous reduction of FM and a tendency to increase peak power. However, it seems obvious that the efficacy of HMB in stimulating an increase in FFM and anaerobic capacity may be observed particularly in the case of incorporation of resistance exercise and/or high-intensity exercise to the training regime, stimulating, to a greater extent, muscle protein synthesis pathways, which would enhance the potential anticatabolic role of HMB.

When assessing the effect of HMB supplementation in endurance sports, the effect of this preparation on aerobic adaptation seems to play a key role. Vukovich and Dreifort [24], after a 2-week HMB supplementation in cyclists, recorded an increase (*p* < 0.05) in peak oxygen uptake ($\dot{\mathrm{V}}{\mathrm{O}}_2\ \mathrm{peak}$) by 4 % and an extension of the time required to reach $\dot{\mathrm{V}}{\mathrm{O}}_2\mathrm{peak}$ by 3.6 %. Moreover, values of these indices increased (*p* < 0.05) also in comparison to the results recorded in groups administered leucine or a placebo. Similar results were observed in this study, in which $\dot{\mathrm{V}}{\mathrm{O}}_2 \max$ of rowers increased after 12-week HMB supplementation, both in relation to the placebo treatment and values before its supplementation (Pre_HMB_). In the study by Vukovich and Dreifort cited above, the administration of HMB also led to an advantageous increase in the lactate threshold, which, when expressed in percent $\dot{\mathrm{V}}{\mathrm{O}}_2\mathrm{peak}$, increased by 8.6 %. Also found was a delayed OBLA observed at the oxygen uptake, which increased by 9.1 %. Thus, these results seem to confirm the effect of HMB supplementation observed in this study on the increase in aerobic adaptation of athletes. Furthermore, these observations correspond also to the latest results reported by Robinson et al. [25], who, in a group of males and females after a 4-week HMB supplementation, combined with high-intensity interval training, found levels of $\dot{\mathrm{V}}{\mathrm{O}}_2\mathrm{peak}$ higher by almost 5.9 % (*p* = 0.032) and 9.8 % (*p* < 0.001), respectively, in comparison to the placebo and the control groups. The authors also found VT to be higher by almost 9.3 % (*p* = 0.017) and 16.5 % (*p* = 0.012), respectively. Another important point showed Lamboley et al. [26], in the previously described study, also showed an advantageous effect of HMB supplementation resulting from the considerable increase in $\dot{\mathrm{V}}{\mathrm{O}}_2 \max$ by as much as 7.7 ml · kg^−1^ · min^−1^. In both groups, a significant improvement was also found in VT (+11.1 % _HMB_ vs. +9.0 % _PLA_). Despite the increase in $\dot{\mathrm{V}}{\mathrm{O}}_2 \max$ values recorded in this study in the group supplemented with HMB, the differences were not high, which suggests that they may have resulted, to a considerable degree, from the fact that participants in this study practiced sports as recreation and, prior to the onset of the experimental procedure, had no aerobic training. In contrast, this study involved elite rowers and, even a slight increase in aerobic capacity in their case, may be considered to be particularly advantageous.

Thus when considering the results of this study, literature data as well as the above mentioned potential mechanisms of HMB action, connected (for example) with the regulation of muscle protein expression, maintenance of cell wall integrity or stimulation of activity of AMPK kinase and Sirt 1, which promotes stimulation of mitochondrial biogenesis, higher oxygen consumption and increased efficiency of carbohydrate, glycogen and fat metabolism [17, 18, 39, 40], it may be inferred that HMB supplementation under specific conditions seems to also enhance the increase in aerobic capacity. Increasing the usability and availability of energy substrates thus appears to explain the growth of aerobic adaptation (VO_2_max, VT) of rowers. Observed after HMB supplementation physical capacity increase, could be in practice due to the more efficient use of exercise stimulus, as well as increase the efficiency of post-exercise recovery period, which are necessary to achieve super compensation.

It turns out that HMB supplementation applied in this study had no effect on the levels of blood biochemical markers. Literature data also did not clearly show the effect of HMB on changes in their concentrations. Nissen et al. [2] and van Someren et al. [22], following HMB supplementation, found a lower activity of CK and/or LDH in the blood of examined individuals. The above observations seem to suggest that HMB supplementation may play a significant role in the reduced rate of muscle damage. However, long-term HMB supplementation in trained individuals, e.g. as a result of homeostatic mechanisms in the organism, may reduce the effects of this substance on the level of adaptation of the organism verified by the analyses of levels of standard biochemical markers in the blood. To confirm this thesis, Gallagher et al. [19], in a group receiving HMB, showed a lower CK activity (by approximately 200 U · kg^−1^) 48 h after a series of resistance exercises; however, this effect disappeared after a longer supplementation period. In turn, in runners, Knitter et al. [20] observed lower concentrations of CK and LDH in a group supplemented with HMB immediately after they completed a 20 km race, as well as during the three successive days after this exercise. Thus, the cited studies seem to confirm the hypothesis on the effect of HMB on the stimulation of sarcolemma integrity and inhibition of proteolytic activity of the ubiquitin-proteasome system. This may indicate the advisability of HMB supplementation in sports due to the reduced rate of muscle damage as a consequence of intensive exercise loads.

It’s important to observe that a limited number of studies analysed the effect of HMB uptake on the systemic hormone metabolism. In comparison to the resting-state hormone concentrations recorded prior to the tests and following 12 weeks of HMB administration combined with power training, Kreamer et al. [41] showed a significant increase in the pre-exercise concentration of testosterone and a reduction of cortisol levels, which were not observed in the control. In supplemented group 15 min after the completion of exercise, blood testosterone concentration increased considerably, but after 30 min, the level of this hormone was similar to that recorded in the control group. No significant differences were observed in the blood concentrations of cortisol, although in this supplemented group a reduced level 30 min after exercise was found. In a recent paper of Townsend et al. [42] testosterone levels significant increased immediately after exercise in comparison to baseline, but also returned after 30 min, in resistance trained men, supplemented with HMB. This finding might explain no significant results observed in this study. Seems to be possible that HMB supplementation can promotes hormonal changes observed in a time-dependent manner.

We would like to highlight here that numerous studies are consistent with the results of this study and do not confirm the effect of HMB, in comparison to placebo, on activity of CK and LDH [19, 27–29, 43] or blood testosterone concentration [3, 27, 28]. We need to mention here that when assessing levels of biochemical markers following supplementation, it is difficult to reliably compare the presented studies. The final results may have been affected not only by the dose of the preparation, but also by the duration of supplementation, the training standard of athletes involved in the experiment and applied training loads.

## Conclusions

This study indicates that HMB supplementation in athletes training for endurance sports promotes the advantageous increase in aerobic capacity of the organism, mainly due to the increased values of maximum oxygen uptake and indices of the VT, as well as the reduction of fat mass. It may also enhance peak anaerobic power. Long-term HMB supplementation seems not only to have a significant effect on changes in activity of selected intramuscular enzymes testosterone and cortisol concentration, but also on values of the T/C ratio in blood.

## Abbreviations

AMPK kinase: Adenosine monophosphate-activated protein kinase
AP: Average power
BM: Body mass
CK: Creatine kinase
FFM: Fat-free mass
FM: Fat mass
HMB: β-hydroxy-β-methylbutyric acid
HRmax: Maximum HR
HR_VT_: HR at VT
LDH: Lactate dehydrogenase
MP: Minimal power
mTOR kinase: Mammalian target of rapamycin kinase
$\dot{\mathrm{V}}{\mathrm{O}}_2 \max$: Maximal oxygen uptake
$\dot{\mathrm{V}}{\mathrm{O}}_2\mathrm{peak}$: Peak oxygen uptake
PLA: Placebo
PP: Peak power
Sirt 1: Sirtuin 1
T/C ratio: Testosterone/cortisol ratio
Tref: Exercise time to athlete’s refusal to continue exercising
Wmax: Maximum load
T_VT_: Time to VT
VT: Ventilatory threshold
W_VT_: Load at VT
